# Contrast flow pattern in the space of Okada during fluoroscopic-guided interlaminar lumbar epidural injection

**DOI:** 10.1016/j.inpm.2024.100530

**Published:** 2024-11-29

**Authors:** Yakov Vorobeychik, Almeet Kaur

**Affiliations:** Penn State Health Milton S Hershey Medical Center, Department of Anesthesiology and Perioperative Medicine, 500 University Dr. Hershey, PA, 17036, United States; Penn State Health Milton S Hershey Medical Center, Department of Physical Medicine and Rehabilitation, 500 University Dr. Hershey, PA, 17036, United States

Dear Editor:

The space of Okada was first identified in the cervical spine by Kikuzo Okada in 1981 during an anatomical study of cervical facet joints. The study found a communicating pathway dorsal to the ligamentum flavum in 80 % of the 142 joints, linking the facet joint to the interlaminar and interspinous portions, contralateral facet joint, and cervical extradural space [1]. The space of Okada is limited to the interlaminar region and is not continuous in the coronal plane. It is rarely observed in the lumbar spine, except in cases of pars interarticularis defects, Baastrup disease, or recently described posterior ligamentous complex inflammatory syndrome [[2], [3], [4]].

Several case reports and case series have documented unintended injections into the space of Okada during attempted transforaminal and interlaminar epidural steroid injections, as well as intraarticular facet joint injections [[5], [6], [7], [8], [9], [10], [11]]. Studies examining contrast flow during CT- and fluoroscopic-guided cervical interlaminar epidural steroid injections have identified a 2.9%–6.0 % rate of unintended injections into this retrodural space [12,13]. A similar frequency of aberrant contrast flow in the space of Okada, ranging from 0.6 % to 7.5 %, has been reported in lumbar region injections [[14], [15], [16], [17], [18]]. We describe a unilateral contrast flow pattern during interlaminar epidural steroid injection and recommend corrective actions for interventionalists to ensure proper medication delivery into the epidural space.

After encountering loss of resistance to air during an L4-5 epidural interlaminar steroid injection, 1 ml of Omnipaque 240 was injected. A conventional anterior-posterior fluoroscopic view revealed no contrast material in the epidural space. Instead, a small collection of dye was observed in the space of Okada, along with opacification of the entire ipsilateral L4-5 facet joint, extending into the superior and inferior capsular recesses (Fig. 1A). The contrast medium injected into the space of Okada typically spreads bilaterally, creating a characteristic “butterfly" or “mustache” appearance. However, in this instance, only a unilateral dye flow occurred. Both contralateral oblique (CLO) and lateral views confirmed the contrast filling of the space of Okada and L4-5 facet joint (Fig. 1B & C), but no contrast agent in the epidural space could be observed. It is important to note that the lateral view in this case is redundant and could potentially be misleading, as the tip of the needle is not positioned strictly in the midline and may appear more ventral than its actual location. As demonstrated in the seminal studies by Gill et al., the contralateral oblique view is superior to the lateral view for accurately assessing needle positioning [19,20]. The distribution of contrast material dorsal to the ventral interlaminar line (VILL) clearly indicates the extradural location of the needle.

**Fig. 1:**
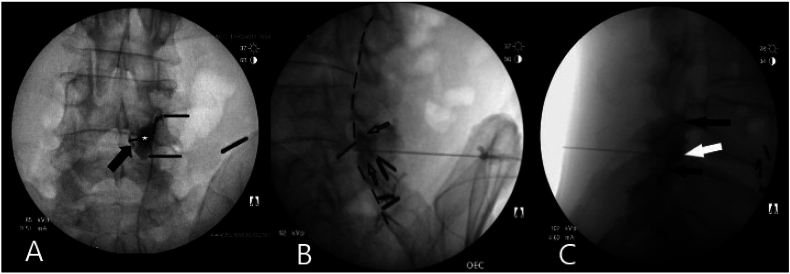
Contrast flow pattern in the retrodural space of Okada after encountering “false” loss of resistance during L4-L5 interlaminar lumbar epidural steroid injection. **A:** Anterior-posterior view shows a collection of contrast medium in the space of Okada (thick black arrow) spreading into the L4-5 facet joint (white star) and extending into its superior and inferior capsular recesses (thin black arrows.) **B:** Contralateral oblique view reveals the contrast flow in the space of Okada, filling the entire L4-5 facet joint (outlined arrows). No contrast material is observed anterior to the ventral interlaminar line (dashed line), indicating no dye spread into the epidural space. **C:** Lateral view shows opacification of the L4-5 joint (black arrows) and a denser blob of contrast medium at the tip of the needle (white arrow), where a collection of dye in the space of Okada is superimposed on the dye flow inside the facet joint.

Further advancement of the needle led to regaining the feeling of the resistance exerted by ligamentum flavum and, with continuing advancement, to the second “true" loss of resistance. An additional bolus of contrast medium injected at that point showed a typical irregular, lobulated epidural flow pattern punctuated by “vacuoles" when it surrounds locules of epidural fat (Fig. 2 A). Despite this, the appearance of the previously injected contrast material in the space of Okada and facet joint remained unchanged.

**Fig. 2:**
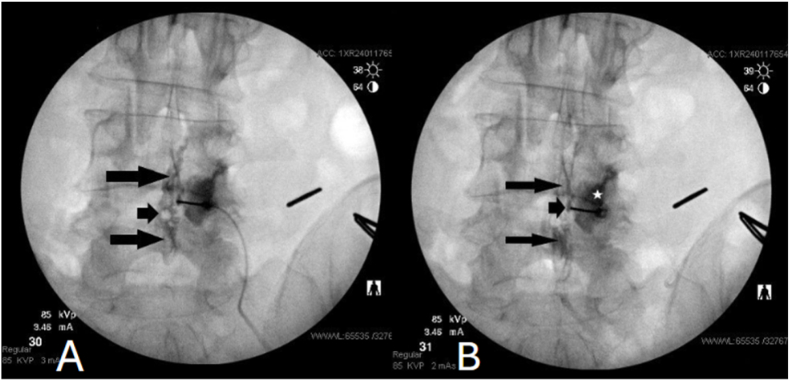
**A:** Contrast flow pattern in the epidural space following the identification of a “true” loss of resistance during the same injection. The L4-5 facet arthrogram and a small collection of contrast medium in the space of Okada have not changed from previous observations. However, the additional injection of dye reveals a typical epidural flow pattern (long black arrows). The contrast medium exhibits irregular pattern interspersed with patchy vacuolization (short black arrow). **B:** Contrast flow pattern subsequent to the injection of a therapeutic solution. Anterior-posterior radiograph fluoroscopic image demonstrates a “washing out” of the contrast material in the epidural space (long black arrows). The shape and density of the dye collection in the facet joint (white star) and the space of Okada (short black arrow) remain unchanged.

At that point, the operator felt confident that the needle was accurately placed in the epidural space and proceeded to inject the therapeutic solution without obtaining additional lateral or contralateral oblique views. A post-injection anterior-posterior radiograph revealed a “washing out" of the dye in the epidural space but not in the space of Okada or facet joint (Fig. 2 B).

Unlike previous studies that demonstrated bilateral contrast distribution within the space of Okada and the facet joints, the radiographs presented here illustrate an ipsilateral spread of dye on the anterior-posterior images. The needle tip is positioned near the VILL in the CLO view, indicating a potential defect in the ligamentum flavum. This defect may allow communication with the ipsilateral facet joint through the space of Okada.

It is crucial for interventional pain practitioners to be able to identify the aberrant contrast flow pattern in the retrodural space of Okada during fluoroscopic-guided epidural steroid injections. They should be prepared to make necessary adjustments to ensure that the medication is accurately delivered into the epidural space. Various abnormal contrast flow patterns (such as intrathecal, intradural, or vascular flow) may be observed during interlaminar epidural steroid injections. If these patterns are recognized, the procedure may need to be stopped, or the needle may need to be repositioned significantly in order to reach the epidural space. However, if the space of Okada is accidently accessed during the fluoroscopic-guided interlaminar epidural steroid injection, simply advancing the needle ventrally will guide it into the intended epidural space.

## Declaration of competing interest

The authors declare that they have no known competing financial interests or personal relationships that could have appeared to influence the work reported in this paper.
